# Distinct Activity Profiles of Somatostatin-Expressing Interneurons in the Neocortex

**DOI:** 10.3389/fncel.2017.00273

**Published:** 2017-09-11

**Authors:** Srikanth Ramaswamy, Cristina Colangelo, Eilif B. Muller

**Affiliations:** Blue Brain Project, Ecole Polytechnique Fédérale de Lausanne, Campus Biotech Geneva, Switzerland

**Keywords:** neocortex, interneurons, pyramidal cells, somatostatin, vasoactive intestinal polypeptide, acetylcholine, channelrhodopsin, optogenetics

A distinct characteristic of the mammalian neocortex is the daunting diversity of inhibitory interneurons. Inhibitory interneurons must be reliably activated to maintain the crucial balance between excitation and inhibition, and are therefore, essential for faithful information processing in the neocortex. In the neocortex, inhibitory interneurons can be distinctly classified into electrical, anatomical, and molecular types that play specific roles in orchestrating network activity. In the midst of the overwhelming assortment of neocortical interneuron types, however, Somatostatin (Sst) expressing neurons emerge among the relatively well characterized (Urban-Ciecko and Barth, 2016). Sst interneurons are quite ubiquitous in their occurrence in neocortical microcircuitry, and are found across both infragranular and supragranular layers (Wang et al., 2004; Yavorska and Wehr, 2016). Depending on the brain region, subtypes of Sst interneurons can differentially express other calcium-binding proteins, neuropeptides and enzymes such as calbindin, calretinin, neuropeptide-Y (NPY), cholecystokinin, and nitric oxide synthase (Dun et al., 1994; Gonchar and Burkhalter, 1997; Kawaguchi and Kubota, 1997; Kubota et al., 2011). More often than not, Sst and NPY are found to be co-expressed, and are also known to occur along with calbindin in the same group of interneurons (Wang et al., 2004; Gonchar et al., 2008; Urban-Ciecko and Barth, 2016). These differential patterns of co-expression suggest that despite being located in the same brain region, subtypes of Sst interneurons can be functionally unique. Sst interneurons are implicated in a spectrum of neurological disorders including schizophrenia, epilepsy, depression, and Alzheimer's disease (Burgos-Ramos et al., 2008; Morris et al., 2008; Zhang et al., 2009; Fuchs et al., 2017). Therefore, understanding the anatomy and physiology of Sst interneurons is not only vital for brain function but also to identify therapeutic targets in dysfunction.

In comparison with other interneuron types, Sst neurons stand out by virtue of a striking axon that ascends toward and arborizes extensively in layer 1, where it inhibits pyramidal cells (PCs) by directly innervating their distal apical dendrites and terminal tufts (Markram et al., 2004; Silberberg and Markram, 2007). Sst axons originating from supragranular layers can also innervate PCs in neighboring and distant neocortical microcircuits, providing the only source for cross-columnar inhibition via layer 1 (Ma et al., 2006). Sst neurons are not only unique in their axonal morphology, but also display a conspicuous action potential (AP) discharge pattern, which is pronounced by slow spike-frequency accommodation and a low threshold for AP initiation (Kawaguchi and Kubota, 1996; Goldberg et al., 2004; Wang et al., 2004; Ma et al., 2006; Fanselow et al., 2008). Paired whole-cell recordings in brain slices have demonstrated that Sst neurons are firmly embedded into neocortical microcircuitry, and therefore, strongly inhibit other excitatory and inhibitory neurons located within the same or across different cortical layers (Beierlein et al., 2003; Kapfer et al., 2007; Silberberg and Markram, 2007; Berger et al., 2009; Xu et al., 2013). Sst neurons connect to neighboring PCs in neocortical microcircuitry with a high probability of about 30–40%, and receive inputs from adjacent PCs with a probability of about 10–20% (Silberberg and Markram, 2007). Surprisingly, while Sst neurons can establish chemical synapses with parvalbumin (PV)-expressing or vasoactive intestinal polypeptide (VIP)-expressing interneurons in their immediate vicinity, they selectively avoid forming connections with neighboring Sst neurons (Pfeffer et al., 2013). However, Sst neurons could form electrical synapses with one another (Gibson et al., 1999; Beierlein et al., 2000, 2003). In general Sst neurons receive strongly facilitating excitatory inputs from PCs and provide robust depressing inhibitory outputs to PCs, PV, and VIP-expressing neurons (Markram et al., 1998; Silberberg and Markram, 2007; Pfeffer et al., 2013). Neocortical Sst interneurons disinhibit PCs by inhibiting PV-expressing interneurons, suggesting an important circuit mechanism for the balance between excitation and inhibition (Pfeffer et al., 2013; Xu et al., 2013). However, in animal models of neurodegenerative diseases such as amyotrophic lateral sclerosis and frontotemporal dementia, Sst neurons lose their critical ability to disinhibit PCs, indicating that impairments in Sst-mediated inhibition causes the hyperexcitability, excitotoxicity, and degradation of PCs (Zhang et al., 2016).

Much of our understanding of the anatomy and physiology of Sst neurons and their selective inhibition of the distal dendrites and tufts of PCs comes from whole-cell recordings in cortical slices (Wang et al., 2004; Ma et al., 2006; Pérez-Garci et al., 2006; Silberberg and Markram, 2007; Berger et al., 2009; Xu et al., 2013; Ramaswamy and Markram, 2015; Urban-Ciecko et al., 2015). A relatively small number of studies have focused on unraveling the properties of Sst neurons in behaving animals, mostly in the supragranular layers (Murayama et al., 2009; Gentet et al., 2012; Tremblay et al., 2016; Veit et al., 2017). However, technical limitations have hindered direct measurements of the physiology of Sst neurons in the infragranular layers during behavior. To fill this gap, a recent study published in the journal *Science* by Muñoz and colleagues combined optical-tagging and whole-cell recordings to specifically target Sst neurons in both supragranular and infragranular cortical layers in behaving mice (Muñoz et al., 2017). Muñoz et al. used channelrhodopsin-2 (ChR2)-assisted patching, a high-yield method that enables the anatomical and physiological dissection of neurons at any depth of cortical tissue *in vivo* to record from genetically labeled Sst neurons across all cortical layers (Muñoz et al., 2017). The authors then leveraged this strategy in awake *SstCre* mice that were crossed to a reporter line expressing ChR2-enhanced yellow fluorescent protein (EYFP) to label and record from light-activated Sst neurons in all layers of rodent somatosensory cortex. Muñoz et al. discovered that functionally distinct Sst neuron subtypes located in specific cortical layers with specialized axonal innervation patterns were activated in contrasting ways during whisking behavior. The authors further found that the activity of Sst neurons *in vivo* was regulated by the relative strength of inhibition received from VIP-expressing interneurons and cholinergic neuromodulation.

In a first set of experiments, Muñoz et al. used ChR2-assisted patching in order to relate Sst neuron activity to behavioral state by tracking periods of quiescence and whisking activity through whisker pad electromyogram recordings and local field potentials (LFPs) at different cortical depths. The authors found that the recorded activity of a major proportion of Sst neurons was significantly modulated during whisking behavior and were accompanied by transitions to cortical network activation as defined by decreased power of low frequencies and increased power of high frequencies in the LFP. During active whisking, the authors discovered that Sst neurons located in layer 2/3 suppressed their firing activity (termed Wh_OFF_). However, during the same whisking paradigm Sst neurons in layer 4 differed strikingly from their counterparts in layer 2/3 by enhancing their spiking pattern (called Wh_ON_), which revealed disparate activity modes of Sst neurons in the infragranular cortical layers. Venturing into the supragranular layers, Muñoz et al. found that the distribution of Sst neurons based on their spiking response to a whisking behavior as before was again heterogeneous. While upper layer 5 (layer 5A) contained an equal number of Wh_ON_ and Wh_OFF_ Sst neurons, the proportion of Wh_ON_ in layers 5B and 6 was higher. Because the Wh_ON_ and Wh_OFF_ spiking activity patterns were tightly correlated with similar whisking attributes and LFP frequency properties, the authors could ascertain that these differences did not result from changes in the level of alertness of the animals.

Sst neurons in different layers of the neocortex are characterized by conspicuous axonal arbors (Wang et al., 2004; Ma et al., 2006). The authors hypothesized that the heterogeneous distribution of Wh_ON_ and Wh_OFF_ activity patterns across cortical layers demonstrated distinct Sst neuron subtypes. To test their hypothesis, the authors labeled *in vivo* a subset of Wh_ON_ and Wh_OFF_ Sst neurons and reconstructed their morphology. Further analyses of their axonal branching pattern revealed five unique Sst neuron subtypes localized in specific cortical layers. Sst neurons in supragranular layer 2/3, exclusively Wh_OFF_ neurons, were archetypal Martinotti cells with axons ascending to layer 1 and densely innervating layers 1 and 2/3, where they provide inhibition to the terminal tufts of pyramidal cells originating in layers 2/3 and 5. As opposed to layer 2/3 Sst neurons, their counterparts in layer 4 were all Wh_ON_ cells and classified as non-Martinotti cells because their axons did not ascend to layer 1 but were confined to layer 4, where they typically innervate PV-expressing neurons (Muñoz et al., 2017). In infragranular layers 5A and B, Wh_OFF_ Sst neurons were found to be Martinotti cells with local axonal collaterals contained in layer 5A and a “T-shaped” translaminar axon terminating in the uppermost part of layer 1. Contrastingly, Wh_ON_ Sst neurons in layer 5, which were again Martinotti cells had broad ascending axons that “fanned-out” across lower layer 1 and upper layer 2/3. The somata and local axonal plexus of Wh_ON_ Martinotti cells were situated deeper in layer 5 compared against Wh_OFF_ Martinotti cells. The final subset of Wh_ON_ Sst neurons in layers 5A and B were identified as non-Martinotti cells with their somata and local axonal arbor contained in layer 5B/6 and ascending axons densely innervating layers 3 and 4. Due to the dissimilarity in the innervation realm of Wh_ON_ and Wh_OFF_ Sst neurons in infragranular layers, inhibition is diverted onto layer 5 pyramidal cells during active whisking behavior, with their apical and oblique dendritic compartments in layer 2/3 receiving increased inhibition and terminal tufts confined to the upper parts of layer 1 obtaining decreased inhibition. Based on these observations, Muñoz et al. predict that inhibition and disinhibition cooperate with a layer-specific organization of excitation on the exquisite dendritic branches of layer 5 pyramidal cells (Muñoz et al., 2017).

Muñoz et al. then asked what cellular and network mechanisms could shape the activity of Martinotti and non-Martinotti Sst neurons during active whisking behavior? Previous work has identified that VIP-expressing interneurons suppress the activity of layer 2/3 Sst neurons in behaving animals (Xu et al., 2013). But, the finding by Muñoz et al. that a bulk of Sst neurons in layers 4 and 5 are show Wh_ON_ activity profiles contradicts established roles of VIP-expressing interneurons. However, it is known that the axons of VIP interneurons strongly innervate layer 2/3 and 5A (Tremblay et al., 2016). The authors, therefore, hypothesized that laminar differences in the innervation of Sst neurons by VIP-expressing interneurons could lead to differential control of the activity of Sst neurons *in vivo*. To test their hypothesis, Muñoz et al. used double-transgenic mice to label the axons of VIP-expressing interneurons with ChR2 and record light-evoked inhibitory postsynaptic responses in Sst neurons *in vitro*. The authors discovered that VIP interneurons provided stronger inhibition to Sst neurons located in layer 2/3 as against those found in layers 4 and 6. Exceptionally, in the infragranular layers only Sst neurons situated in layer 5A were strongly inhibited by VIP interneurons, which was consistent with the laminar localization of Wh_OFF_ Sst neurons in the infragranular layers (Muñoz et al., 2017). Indeed, T-shaped Martinotti Sst neurons that were analogous with Wh_OFF_ activity *in vivo* produced light-evoked inhibitory postsynaptic responses of higher amplitudes in comparison with Wh_ON_ Sst neurons that were either fanning-out Martinotti or non-Martinotti cells. Importantly, differences in the strength of inhibitory postsynaptic responses were significantly correlated with the number of appositions from the axons of VIP neurons onto the dendrites of Sst neurons. While the innervation pattern of appositions from VIP axons onto T-shaped and layer 2/3 Martinotti cells culminated proximally at the soma, appositions were distributed more distally in fanning-out Martinotti and non-Martinotti cells.

Next, Muñoz et al. tested if the Wh_OFF_ activity profile of layer 2/3 and T-shaped Martinotti cells observed *in vivo* resulted due to inhibition from VIP neurons. To test this, the authors expressed a pharmacogenetic catalyst, which suppresses the excitability of VIP neurons upon activation of its synthetic ligand. Using a combination of ChR2 expression and histological methods, Muñoz et al. then dampened the activity of VIP neurons *in vivo* that consequently abolished inhibition onto Wh_OFF_ Sst neurons and flipped them into Wh_ON_ Sst neurons. This perturbation did not significantly impact the activity of Wh_ON_ Sst interneurons and corroborated their feeble inhibition by VIP neurons, thus demonstrating a mechanistic link between the excitability of VIP neurons and their regulation of Wh_OFF_ Sst neurons (Muñoz et al., 2017).

The authors then set out to identify the potential factors driving the activity of Wh_ON_ Sst interneurons during behavior. It is known that the primary somatosensory cortex receives excitatory input from the thalamus and primary motor cortex (Petreanu et al., 2012; Poulet et al., 2012). Although Sst neurons are also targeted by these sources, the excitation they receive is weak. However, Sst neurons receive powerful depolarizing input through the activation of muscarinic receptors *in vitro*, and cholinergic modulation of neocortical activity is related to active behavior such as whisking and locomotion (Eggermann et al., 2014; Nelson and Mooney, 2016). Therefore, Muñoz et al. hypothesized that acetylcholine could drive the activity of Sst interneurons *in vivo* and tested the role of muscarinic receptors in modulating activity profiles of Sst neurons during whisking. Local application of atropine, a common muscarinic antagonist, suppressed or even flipped the activity of Wh_ON_ Sst interneurons, and further quietened the activity Wh_OFF_ cells. Because acetylcholine differentially regulates the physiology of neocortical neuron types and their synapses (Levy et al., 2008; Muñoz and Rudy, 2014), the authors probed whether cholinergic modulation of Wh_ON_ Sst neurons *in vivo* was a generic effect or independent of cell-type in mutant mice where M1 and M3 muscarinic receptors were genetically knocked-out in Wh_ON_ Sst neurons. The authors validated that the number and distribution of neocortical Sst interneurons across different layers, and their physiological features in mutant mice were similar to those seen in wild-type mice. Subsequent experiments in brain slices confirmed that the activity of Sst neurons in mutant mice was unaffected by muscarinic receptors. In a next set of experiments in mutant mice, Muñoz et al. expressed ChR2 in Sst neurons and recorded activity *in vivo* from a subset of these neurons across layer 2 to 6 where M1 and M3 muscarinic receptors were genetically ablated. The authors discovered that with the exception of a single Sst neuron, the remainder of mutant Sst neurons displayed Wh_OFF_ activity profiles compared to the activity of wild type Sst neurons under similar experimental conditions. Mutant Wh_OFF_ Sst interneurons were located in layers that predominantly displayed Wh_ON_ profiles. Muñoz et al. thus deduced that during active whisking Sst interneurons are strongly excited by cholinergic modulation through through M1 and/or M3 muscarinic receptors, which is a crucial factor for Wh_ON_ activity.

In a final set of experiments, the authors sought to address whether Sst neurons also preserve their functional novelty during quiet wakefulness or non-whisking states. Quiet wakefulness is related to the delta (about 1–4 Hz) and gamma (around 40–100 Hz) frequency oscillations of the neocortical LFP due to changes in the excitability of cortical networks. Therefore, Muñoz et al. studied the relationship between the firing activity of Wh_ON_ and Wh_OFF_ Sst neurons and the properties of delta and gamma frequency LFP bands during quiet wakefulness or non-whisking states. Interestingly, the firing rate of Wh_ON_ Sst neurons correlated more strongly with the LFP of the delta-band activity than that of Wh_OFF_ Sst interneurons. In addition, the firing activity of separate Sst neuron subtypes was linked to different phases of delta oscillatory cycles, and phase coupled to gamma oscillatory cycles.

This study by Muñoz et al. demonstrates the layer-specific modulation of Sst neurons in somatosensory cortex where their firing activity increases or decreases during active whisking. The increase or decrease in firing activity was further related to the axonal morphology of Sst neurons. An important finding of this study is that Sst neurons are differentially inhibited by VIP neurons on one hand and excited by cholinergic modulation on the other. This provides evidence for a push-pull switching mechanism balancing the yin of inhibition and the yang of excitation, which regulates Wh_ON_ and Wh_OFF_ activity profiles in disparate subtypes of Sst neuron. While this study lays a foundation for identifying key roles for specific types of inhibitory interneurons during active behavior, several questions remain to be addressed. An important question is whether the correlation between Wh_ON_ and Wh_OFF_ activity profiles and the axonal architecture of Sst neurons in supragranular and infragranular layers can be ubiquitous to other cortical areas? For example, it is known that the morphology of pyramidal cells progressively increases in complexity from the occipital and temporal lobes to the parietal and frontal lobes (Ramaswamy and Markram, 2015). It is yet to be quantified, however, whether Sst neurons could follow suit. Hypothesizing that they do, not only will their inhibition of pyramidal cells change by virtue of compartmentalized dendritic innervation to differentially impact the initiation sites of local regenerative events such as N-methyl-D-aspartate (NMDA) and Ca^2+^ spikes, but also their Wh_ON_ and Wh_OFF_ profiles could be drastically altered from the occipital to frontal lobes. Extending the authors' study to record from Sst neurons in other parts of the neocortex during active behavior could test these hypotheses.

It must be noted that *in vitro* slice recordings by Muñoz et al. were performed at 2 mM extracellular Ca^2+^. However, since extracellular Ca^2+^ *in vivo* is about 1.2 mM it would be important to ascertain the amount by which the observed physiology of VIP neuron-mediated inhibitory responses could change in 1.2 mM extracellular Ca^2+^ as against 2 mM extracellular Ca^2+^. While Muñoz et al. point out that VIP neurons provide a critical source of inhibition by differentially innervating Sst neuron subtypes either perisomatically or distally, their study crucially omits to provide morphological reconstructions of VIP neurons. In the neocortex, bipolar, double bouquet and small-basket cells, all of which predominantly express VIP, subserve diverse functions and have different axonal and dendritic architectures (Markram et al., 2004). Therefore, it is essential to peel out the morphologies of local VIP neurons that differentially inhibit Sst neurons to better understand their global role at the network level. Muñoz et al. show that cholinergic modulation is an important driver of the Wh_ON_ Sst interneurons during behavior. Previous work has shown that bipolar cells in layer 2/3 of the neocortex coexpress VIP and acetylcholine (von Engelhardt et al., 2007). Although cholinergic input to the neocortex mostly originates in the basal forebrain the authors would need to ascertain, however, if the VIP neurons identified in this study also coexpress and corelease acetylcholine. This provides an interesting hypothesis to test—could VIP neurons be the sole master circuit switch providing the push-pull mechanism controlling the activity of Wh_ON_ and Wh_OFF_ Sst neuron subtypes?

Muñoz et al. used mice lines where both M1 and M3 muscarinic receptor sub-types were genetically knocked-out to establish their involvement in modulating Wh_ON_ Sst neurons. However, the contribution of nicotinic receptor sub-types in controlling Wh_ON_ activity remains to be fully ascertained. A recent study in the primary visual cortex demonstrated that local application of ACh evoked robust depolarization in Sst neurons, which persisted in the presence of glutamatergic and GABAergic antagonists, but was significantly reduced in the presence of specific muscarinic and nicotinic antagonists such as atropine and mecylamine, respectively (Chen et al., 2015). Chen et al. further corroborated by immunohistochemistry that both muscarinic and nicotinic receptors are effectively expressed in Sst neurons, and confirmed that Sst interneurons are directly activated via both types of receptors (Chen et al., 2015). Therefore, future experiments should consider the possibility of a nicotinic component in regulating the activity of Wh_ON_ Sst neurons.

In conclusion, the study by Muñoz et al. employs innovative techniques to characterize Sst neurons during behavior and reveals important functions of these neurons in maintaining cortical activity. However, further work is needed to extend the methods developed in this study to build a unifying view of the roles of other inhibitory interneuron types in the neocortex in processing sensory information, and in particular how they are differentially regulated by the “big-5” neuromodulators—histamine, acetylcholine, noradrenaline, dopamine, and serotonin. Such studies could be complemented by recent efforts to build detailed digital models of neocortical microcircuitry to unravel the dynamic function of inhibitory interneurons in shaping cortical network states (Markram et al., 2015; Ramaswamy et al., 2015). Muñoz et al. have identified an essential link toward solving this tantalizing puzzle.

## Author contributions

SR conceived the idea and drafted the manuscript. CC and EM contributed to drafting the manuscript.

### Conflict of interest statement

The authors declare that the research was conducted in the absence of any commercial or financial relationships that could be construed as a potential conflict of interest.
